# Advancements in CAR-NK therapy: lessons to be learned from CAR-T therapy

**DOI:** 10.3389/fimmu.2023.1166038

**Published:** 2023-05-02

**Authors:** Marisa K. Kilgour, Donald J. Bastin, Seung-Hwan Lee, Michele Ardolino, Scott McComb, Alissa Visram

**Affiliations:** ^1^ Cancer Therapeutics Program, Ottawa Hospital Research Institute, Ottawa, Canada; ^2^ Department of Medicine, University of Ottawa, Ottawa, Canada; ^3^ Department of Biochemistry, Microbiology and Immunology, University of Ottawa, Ottawa, Canada; ^4^ Center for Infection, Immunity and Inflammation, University of Ottawa, Ottawa, Canada; ^5^ Human Health Therapeutics Research Centre, National Research Council Canada, Ottawa, Canada; ^6^ Department of Medicine, University of Ottawa, Ottawa Hospital Research Institute, Ottawa, Canada

**Keywords:** natural kill cell, T cell, chimeric antigen receptor, trogocytosis, immunotherapy, cancer

## Abstract

Advancements in chimeric antigen receptor engineered T-cell (CAR-T) therapy have revolutionized treatment for several cancer types over the past decade. Despite this success, obstacles including the high price tag, manufacturing complexity, and treatment-associated toxicities have limited the broad application of this therapy. Chimeric antigen receptor engineered natural killer cell (CAR-NK) therapy offers a potential opportunity for a simpler and more affordable “off-the-shelf” treatment, likely with fewer toxicities. Unlike CAR-T, CAR-NK therapies are still in early development, with few clinical trials yet reported. Given the challenges experienced through the development of CAR-T therapies, this review explores what lessons we can apply to build better CAR-NK therapies. In particular, we explore the importance of optimizing the immunochemical properties of the CAR construct, understanding factors leading to cell product persistence, enhancing trafficking of transferred cells to the tumor, ensuring the metabolic fitness of the transferred product, and strategies to avoid tumor escape through antigen loss. We also review trogocytosis, an important emerging challenge that likely equally applies to CAR-T and CAR-NK cells. Finally, we discuss how these limitations are already being addressed in CAR-NK therapies, and what future directions may be possible.

## Introduction

**Figure 1:**
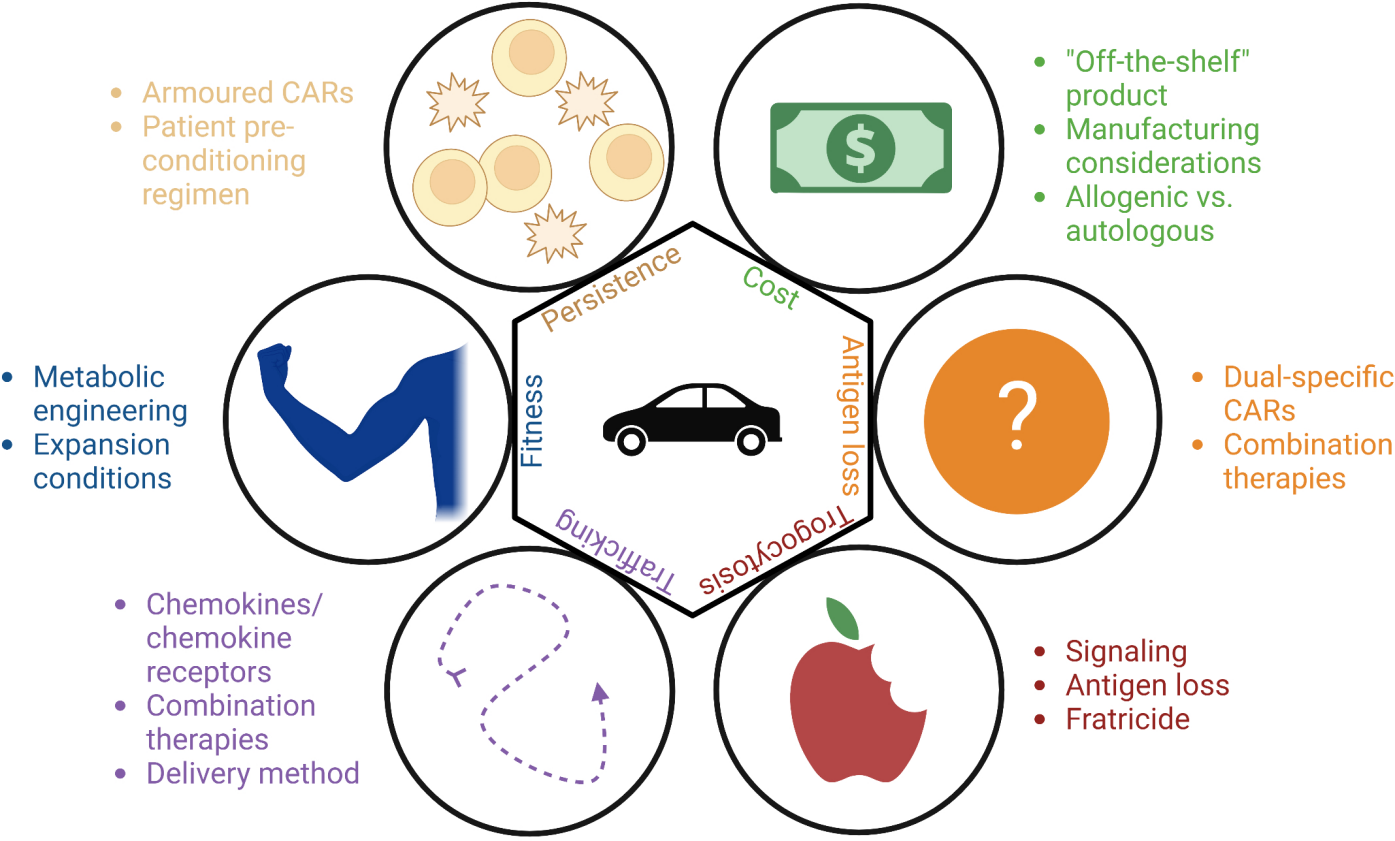
Methods to overcome the barriers to the CAR-T and CAR-NK fields: cost, antigen loss, trogocytosis, trafficking, fitness, and persistence.

**Figure 2:**
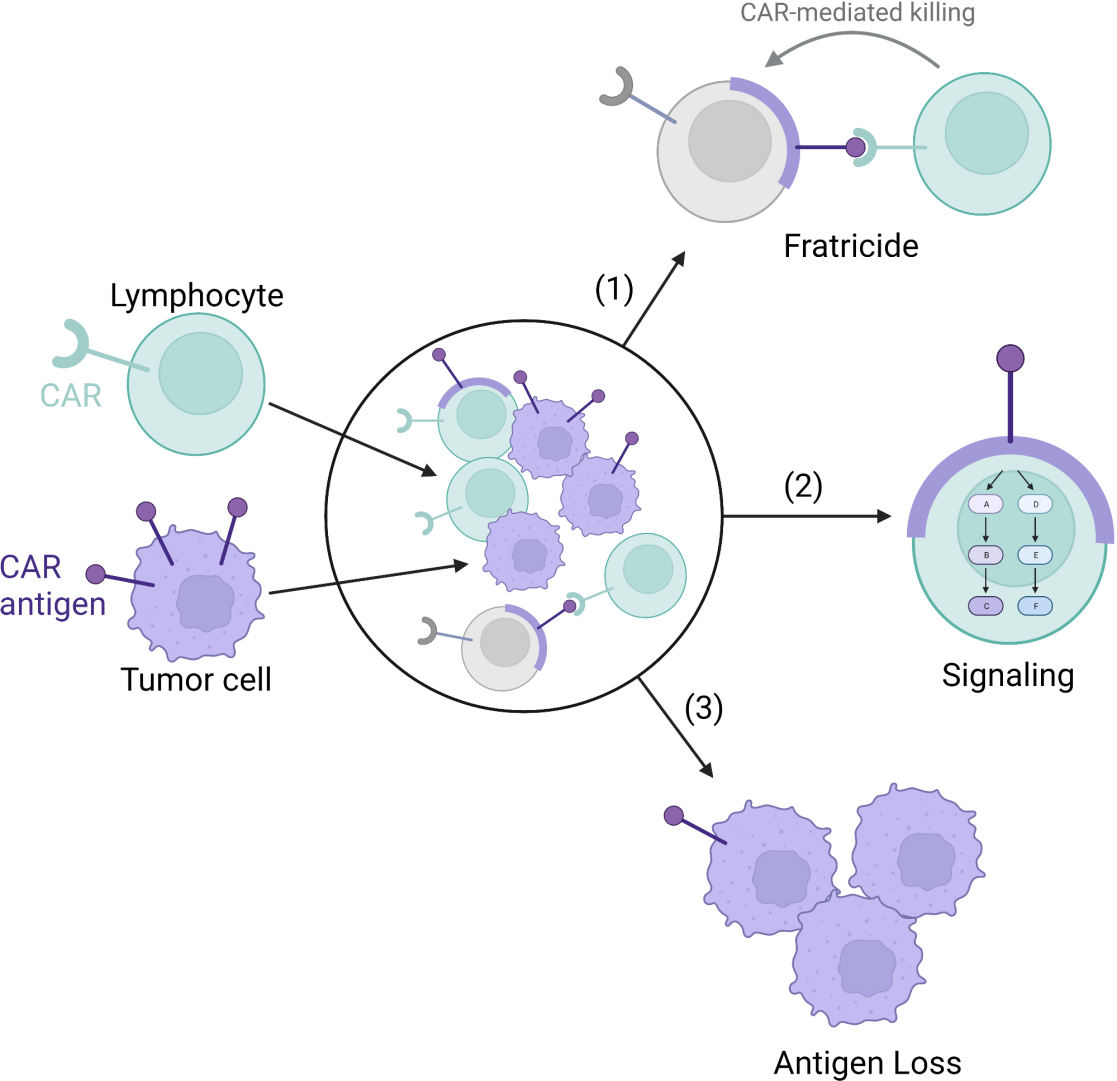
Trogocytosis-mediated antigen procurement leads to trogocytosis-mediated signaling, antigen loss leading to tumor escape, and fratricide. The tumor cell membrane (purple) and CAR antigen is transferred to the CAR-lymphocytes (green) *via* trogocytosis. (1) The transferred membrane acts as a target for CAR-mediated fratricide leading to lymphocyte cell death (grey). (2) The transferred membrane can also signal within the CAR-lymphocyte. (3) Finally, the transfer of the cell membrane results in antigen loss on the tumor cells themselves.

Although chimeric antigen receptor engineered T-cells (CAR-T) have produced astounding remission rates in patients with hematological cancers, response rates have been much lower in patients with solid tumors (1). Furthermore, long-term follow up has shown that approximately half of the patients achieving an initial complete response with CAR-T will ultimately relapse (2, 3). Key barriers leading to suboptimal CAR therapy responses include limited persistence of transferred cells, poor metabolic fitness of cellular products, issues with trafficking of CAR-T cells to sites of disease, and loss of target antigen on malignant cells (**Figure 1**) (4). Another emerging challenge for CAR therapies is trogocytosis, wherein cell membranes are transferred from target cells to immune cells, resulting in antigen loss on target cells and fratricidal destruction of CAR cells (**Figure 2**) (5). Importantly, as clinical experience with CAR-NK is very limited compared to CAR-T, how much each of these factors contribute to CAR-NK clinical efficacy remains unclear. This offers researchers and clinicians a unique opportunity to proactively apply lessons learned while developing CAR-T products to the CAR-NK field. For the remainder of this mini review, we will provide updates on the well-identified barriers and then discuss at length the concept of trogocytosis and the work that has been done to address this increasingly recognized phenomenon.

### Manufacturing and cost

CD19-targeted CAR-T has achieved unprecedented clinical response in leukemia and lymphoma, and now B-cell maturation antigen (BCMA)-targeted CAR-T is yielding remarkable results in patients with multiple myeloma (6, 7). High cost and complex manufacturing for these personalized CAR-T limit access to these therapies (8). Recently, studies have reported the use of distributed and highly automated CAR-T manufacturing as a strategy to significantly reduce manufacturing costs relative to centralized processes (9–11). An alternative cost-reduction strategy would be to develop an “off the shelf” therapeutic, wherein modified cells are manufactured in large numbers at a central location and used allogeneically to treat many patients (12).

Currently, CAR-T products approved for commercial use are all produced using lentiviral or retroviral vector transduction, and are only used autologously due to the risk of graft versus host disease (GvHD). It is important to note though that it may be possible to safely apply donor derived allogenic CAR-T molecules in post-haematopoietic cell transplantion (HCT) settings without acute GvHD (13). Gene editing to remove T cell receptor expression from CAR-T cells has been employed to eliminate the risk of GvHD with TCR-edited allogeneic CAR-T products. However, this increases manufacturing complexity and cost, and the safety profile of such gene-edited CAR-T cells is not yet fully understood (14–16). Unlike CAR-T cells, CAR-NK cells do not cause GvHD and can thus be applied allogeneically without such gene editing (17).

To maximize the benefit of an off-the-shelf therapy, as with gene-edited CAR-T, it will also be vital to identify manufacturing approaches that can scale up CAR-NK products to treat as many patients as possible per product batch (18–20). Like CAR-T cells, cytokines alone can achieve expansion of NK cells to clinically relevant doses (21, 22). NK expansion can be greatly increased with the use of feeder cells, however, feeder cells can be difficult to completely remove from the culture, therefore leading to safety concerns (23–26). An alternative feeder-free method being explored involves the use of dissolveable polymer-based microspheres that slowly release growth factors and nutrients to facilitate cell expansion (27).

Vesicular-stomatitis-virus-G protein (VSV-G) lentivirus is the most commonly used pseudotyping receptor applied in manufacturing CAR-T cells. Unlike T cells though, NK cells have low expression of the low density lipoprotein receptor, a major entry receptor for VSV-G (28). Baboon envelope pseudotyped lentiviral vector (BaEV-LV) has been shown to greatly improve transduction efficiency in NK cells (29), and can also be used for the delivery of CRISPR-genome editing components through viral like particles (30). We have recently shown that CRISPR-loaded BaEV-partcles can be used for efficient simultaneous genome editing of primary NK cells and CAR-transgene delivery, potentially offering a promising opportunity to clinical scale deployment of gene edited CAR-NK therapies (31).

The relative risk of insertional mutagenesis represents another potential issue for CAR-NK therapies manufactured *via* viral gene transfer. There are at least two cases of T-cell lymphomas arising from CAR-modified cells, though it is important to note that both of these occurred in transposon modified cells, a process that can lead to many insertion events within a single cell (32). In contrast to this, thousands of patients have now been treated with CAR-T therapies generated *via* retro- or lentiviral transduction, with follow-up times as long as 20-years, and no malignant T-cell lymphomas have yet been reported (33). There are at least two case reports of insertional disruption of a specific gene being associated with clonal CAR-T hyperexpansion (34, 35). Remarkably, in both cases CAR-T expansion drove a strong therapeutic response without creating a T-cell malignancy. Whether the impressive safety record of virally modified CAR-T cells can also be extended to CAR-NK cells remains to be seen. Publication of pre-clinical and clinical reports with insertion site mapping in CAR-NK cells will be critical to address this question in future.

### Persistence

Dogma dictates that NK cells are inherently less likely to demonstrate long term persistence than T cells (36). Indeed, the short lifespan of transferred cells has been proposed as an explanation for the limited clinical efficacy of NK cell therapies in clinical trials (37–39). To date, much of the work in optimizing CAR constructs for NK cells has focused on improving cytotoxicity as opposed to evaluating persistence (18, 40, 41), though it is known that CAR-NK cell persistence can be improved by engineering cells with immunostimulatory cytokines such as IL-15 (42–49). A clinical trial employing IL-15 engineered CD19-targeted CAR-NK cells in relapsed/refractory hematologic malignancies is currently underway, and has already reported to be safe and show at least short term efficacy in a cohort of patients [(50); NCT05092451].

Short-term exposure to IL-12, IL-15, and IL-18 produces memory-like NK cells with longer persistence (51), which can be combined with CAR-NK to increase efficacy (52). In CAR-T products, expression of certain costimulatory domains is known to enrich for memory phenotype CAR-T cells and is associated with improved persistence and more durable responses in preclinical models and clinical trials (53–55). Engineering of CAR-T cells with immunostimulatory cytokines (secreted or membrane bound) such as IL-7, IL-12, IL-15, IL-18, IL-21, and IL-23, to create “armoured CARs”, has also been shown to improve persistence and is being explored in several clinical trials (56, 57).

Another method to increase CAR cell functionality, safety, and specificity is the use of inducible promoters, which become active upon recognition of a tumor associated antigen, metabolite, drug, or through activation of cell signaling pathways. Ultimately, the goal is to facilitate a more directed delivery of an additional transgene without systemic toxicity (58). This has been shown to be effective in CAR-T cells using a nuclear factor of activated T-cells (NFAT) promoter (59). This type of sense-and-respond engineering of CAR-T cells is extremely flexible and could be applied to deliver a wide variety of TME-modifying payloads to improve CAR-T or CAR-NK penetration and survival within tumours (60, 61). This approach has also been tried with CAR-NK cells; the use of a nuclear factor kappa B (NFκB) inducible promoter to induce IL-12 secretion upon CAR-NK activation led to increased cytotoxicity and monocyte recruitment (62).

A major clinical determinant of NK cell persistence is the lymphodepleting pre-conditioning regimen used to prepare patients before cell infusion. Thusfar, the role of lymphodepletion with CAR-NK therapy has been extrapolated from CAR-T studies, where lymphodepletion has been shown to be vital for the efficacy of CAR-T (63). Typically, a combination of fludarabine/cyclophosphamide is used prior to receiving CAR-NK therapy to reduce NK cell rejection by the host, reprogram the immunosuppressive TME, and decrease tumor burden (18). Comparing high-dose and low-dose lymphodepletion regimens prior to adoptive transfer of unmodified NK cell therapies has demonstrated that high-dose regimens resulted in *in vivo* NK expansion and persistence while low-dose regimens did not (64). The use of CD52-targeting monoclonal antibodies in combination with CD52-knockout CAR-T cells is increasingly being employed as a selective lymphodepletion strategy which can be employed concurrently with cellular infusion (65), whether such an approach can work with CAR-NK cells has not yet been investigated. To our knowledge, there have been no clinical trials comparing lympdepleting regimens in CAR-NK therapies, and this is an area where further investigation will be needed. The need for further study will be even more critical when applying CAR-NK in solid tumours, where the question of the value of lymphodepletion is quite controversial due to the potential for such immune suppression to harm endogenous anti-tumor immune responses.

Ultimately, long-term persistence of CAR-NK cells may not be necessary to achieve long term remissions. While loss of functional CAR-T cells has been established in some clinical trials as the single best predictor of relapse, particularly in the setting of some acute and chronic B-cell leukemias (34, 66), persistence of CAR products may be less important in other malignancies, such as lymphomas (53, 67, 68). In diffuse large B cell lymphoma (a non-hodgkin lymphoma) for example, patients can have durable responses without prolonged CAR-T persistence (67, 69, 70). In line with these observations, optimization of CAR design to maximize short-term or long-term responses appears to be disease specific rather than a one size fits all approach (54). Given this evolution in thinking with regard to CAR-T persistence, it is likely that the need for longer term persistence of CAR-NK cells may similarly be disease specific (53). Clinical studies applying different CAR-NK approaches in different disease settinge will be vital to understand how CAR-NK persistence correlates with outcome. Furthermore, an off-the-shelf CAR-NK therapy could make it easier to compensate for lack of persistence through using multiple infusions to maintain a sufficient number of circulating cells for ongoing disease control.

### Trafficking

Issues with CAR-T or CAR-NK cells in locating and penetrating into the tumor microenvironment (TME) are thought to be an important limit for the efficacy of the therapy in solid tumors (58). One method to improve CAR trafficking is to engineer cells to express chemokine receptors that can directly enhance their ability to track tumor sites. This strategy has been evaluated in pre-clinical studies (71) and is being explored in an early phase clinical trial where CXCR4 co-expression on an anti- B-cell maturation antigen (BCMA) CAR-T was added to increase trafficking to the bone marrow (NCT04727008). Similar to CAR-T cells, NK cells could also benefit from engineered expression of chemokine receptors, as NK cells are known to use chemokine signaling in natural settings (72). NK cells modified to express CXCR2, CXCR4, CCR5, or CCR7 have all been shown to have enhanced tumor control in mice (73–76). In addition, CAR-NK cells engineered to express CXCR1 or CXCR4 also experienced enhanced trafficking to the tumor (74, 77).

Strategies to improve CAR-T trafficking also include attempts to combine with other therapies to make the tumor microenvironment more amenable to lymphocyte recruitment, or modifying the delivery method (direct tumoral injection or systemic delivery) of the CAR therapy (58, 78). The tumor microenvironment can be modified using tools such as oncolytic viruses (OVs) (79), or radiotherapy (80) to increase tumor inflammation (stimulating an immune response) and increase CAR efficacy. While there are substantial studies on combination therapies for CAR-T, combination studies in CAR-NK cells with radiotherapy or OVs are lacking (81). Finally, local injection of CAR-T cells to the tumour, rather than the peripheral blood, improved responses (82–84). This method has also been shown to be safe and efficacious for CAR-NK (85).

### Fitness

For CAR-T therapy to be successful after finding the tumor, CAR-expressing cells need to also survive and function in the harsh TME, where considerable barriers prevent the normal function of immune cells. Barriers include: hypoxia, lack of nutrients, low pH, and elevated levels of various metabolic waste products (86). CAR-T cell products with optimized metabolic functions, such as higher oxidative phosphorylation, have been shown to be more efficacious in the clinic as they can overcome these limitations in the TME (87, 88). Two strategies to metabolically improve T cell function in the TME are: *i)* by direct manipulation of cell metabolism during *ex vivo* expansion, or *ii)* genetically engineering T cells to better cope with the TME (89). As in the case of CAR-T cells, NK cells can also be metabolically optimized during expansion either through genetic alterations or by manipulating expansion conditions (90–93).

Looking at examples of *ex vivo* metabolic engineering of T cells, CD8+ T cells cultured in glutamine restricted conditions had enhanced expression of pro-survival transcription factors and were able to expand better in mice upon reinfusion (94). T cells expanded with mitogen-activated protein kinase kinase (MEK) inhibitors led to decreased tumor burden in mice (95). Finally, T cells were expanded in the presence of phosphoinositide 3-kinase inhibitor (PI3K) to enrich for memory T cells in an ongoing BCMA CAR-T clinical trial which is reported to show promising signs of improved efficacy (96). Even the choice of media can have a major impact on the function and metabolism of the T cells (97). Work in NK cells has been carried out to examine the impact of changing feeder cells used for expansion, and how this can affect metabolic fitness and cytotoxicity (98, 99).

Changes to the CAR itself are also known to alter metabolism, as different signaling domains can significantly change glucose or oxidative metabolism in CAR-T cells (100). Additionally, alteration of the cell by knock-out or overexpression of metabolic genes can increase CAR-T cell efficacy. For example, the TME contains low levels of arginine, so overexpressing enzymes for arginine synthesis in T cells has been shown to improve their efficacy in mouse models of leukemia and solid tumors (101). Similarly, overexpression of D-2-hydroxyglutarate dehydrogenase in CAR-T cells overcomes the immunosuppressive effects of D-2-hydroxyglutarate in tumors with isocitrate dehydrogenase mutation (102). Genetically engineering NK cells to overcome the immune suppressive effects of metabolites, such as hydrogen peroxide through the expression of peroxiredoxin-1 has been shown to improve NK cell function (103). Much opportunity remains to metabolically optimize CAR-NK therapeutics to thrive in the TME.

### Antigen loss

Tumor intrinsic factors also contribute to suboptimal CAR-T responses. This can include immune suppression in the TME through the presence of immunosuppressive cells (e.g. due to cells such as regulatory T cells, myloid derived suppressor cells, tumor associated macrophages, and stromal cells) (104). It can also include resistance due to loss of adhesion molecules on the tumor cells, such as CD58 or ICAM-1 (105, 106), as well as increased expression of apoptotic moleucles (107). The tumor intrinsic factor we will focus on for this section is antigen loss.

Loss of target antigen on tumor cells is an obvious mechanism by which CAR-T therapies fail (108, 109). This may be exacerbated in solid tumors where there is a greater degree of inconsistency in antigen expression (110, 111). Several strategies are under development to circumvent this limitation. Dual-specificity CAR-T cells capable of targeting two separate antigens have achieved strong complete response rates in clinical trials (112–115). Even then, relapses following the loss of at least one antigen have been observed (113–115). Longer follow up and larger trials are needed to determine if these strategies are truly beneficial in reducing relapse and what effect multi-antigen targeting has on CAR-T persistence. Sequential administration of different CAR products has also been explored as a strategy to mitigate tumor antigen loss (116, 117). Furthermore, in preclinical models, combinations with oncolytic viruses, multi-antigen targeting using ankyrin repeat motif CARs, and engineering CAR-T cells to modulate the endogenous immune system are all strategies that have shown promise in limiting relapse by overcoming antigen loss (118–120).

Given the broader antigen independent killing capacity of NK cells, it is possible that CAR-NK cells would have a built-in mechanism for resisting tumor escape due to the loss of CAR target antigen (121, 122). That is, NK cells can rely on their innate killing capabilities rather than on antigen recognition through the CAR alone. However, it is also well established that tumors have the capacity to adapt and evade NK cells both in the context of immunoediting and NK-cell based immunotherapies (123, 124). The implementation of dual target approaches in CAR-NK cells is currently being explored in pre-clinical development (46, 125–128). Clinical experience will show whether loss of multiple antigens is a concern in the context of CAR-NK therapies. However, with either CAR-T or CAR-NKs, identifying better strategies to select the optimal antigen combinations will be critical to creating safe and effective therapies.

### Trogocytosis

The term trogocytosis was used in the 1970s to describe the process in which the amoeba, *Naegleria fowleri*, destroys other cells (129). In 2002, it was adapted to describe the transfer of plasma membrane fragments and associated surface molecules from one cell to another (130). Since then, trogocytosis has been well studied in T and NK cells (131). It has been proposed that trogocytosis provides a mechanism for tissue adaptation of immune cells but can also act as a potent mechanism of immune cell deactivation (132). Trogocytosis has also been documented more recently with CAR-T and CAR-NK cells, causing fratricidal killing and potentially limiting therapeutic efficacy. We will review the consequences of trogocytosis-mediated antigen procurement. Specifically, how this leads to trogocytosis-mediated signaling, target cell antigen loss, and fratricide (**Figure 2**).

#### Trogocytosis-mediated signaling

It has been shown that molecules can be transferred between cells *via* trogocytosis within minutes of contact, and these molecules retain their ability to signal (131), remaining on the cell surface for days (133). Thus, it is important to consider how these acquired proteins change the function of the receiving cell. On one hand, trogocytosis can have positive consequences on NK cell function. For example, NK cells can trogocytose chemokine receptors such as CCR5, CXCR4, or CCR7 (134–136). The acquisition of CCR7 leads to increased NK cell homing to the lymph nodes (135). The acquisition of some molecules, such as TYR03, also increases effector function and proliferation (137). In contrast, trogocytosis can also inhibit NK cell cytotoxicity through the acquisition of immunosuppressive proteins (138–142). In the context of cancer, we found that NK cells acquire PD-1 from leukemia cells (138). In ovarian cancer, NK cells acquire CD9 from ovarian cancer tumor cells, supressing cytotoxicity (139). Despite many studies investigating trogocytosis-mediated signaling, there are few studies proposing methods to counteract these effects. One method to overcome trogocytosis-mediated signaling is to use blocking antibodies against the target antigen. In the case of CD9, NK cell cytotoxicity was restored using a CD9 blocking antibody *in vitro* (139).

To the best of our knowledge, signaling from receptors transferred *via* trogocytosis to CAR-T and CAR-NK cells has not yet been explored. Given that antigens targeted by the CAR can be important signaling molecules in tumor cells (such as BCMA in plasma cells, or mesothelin in solid tumors) (143, 144). Acquisition of target molecules by T or NK cells through trogocytosis may also alter cell function in the CAR cells. Future studies could explore the signaling of trogocytosis-acquired molecules in CAR cells and determine if this is a necessary consideration in designing CAR therapies.

#### Antigen loss

Another consequence of trogocytosis is antigen loss on the target cells. While this has been shown in CAR-NK (145), it is more highly studied in the context of CAR-T (146–148). Since antigen density impacts CAR functionality, downregulation or internalization of the target antigen can lead to tumor escape. Therefore, methods to overcome trogocytosis-induced antigen loss could improve tumor clearance. Trogocytosis may be overcome by tweaking the affinity of the CAR for the target antigen, as low affinity constructs are thought to lead to less trogocytosis while maintaining efficacy (149). Several reports have shown that lower affinity CARs present increased efficacy by preventing tumor escape (147, 149–151); it is tempting to speculate that lower trogocytosis might contribute to this improved function.

Another option to overcome trogocytosis-mediated antigen loss is to adjust the signaling domain of the CAR, as trogocytosis has been shown to affect CARs with CD28 or 4-1BB signaling domain differentially (147). Finally, one interesting study showed that overexpression of cholesterol 25-hydroxylase (CH25H), can lower trogocytosis in CAR-T cells through altered cholesterol metabolism, leading to better outcomes in xenograft models (152). For both T and NK cells, it is not clear at this time whether CAR-expression increases trogocytic transfer by increasing the strength and length of CAR-target cell interaction, or if the antigen-specific binding of the CAR is directly implicated in the process of antigen transfer. More mechanistic studies of trogocytosis in the context of CAR signaling will be needed to better understand the underlying biological processes and how this applies to antigen loss.

#### Fratricide

In some cases, the ultimate consequence of trogocytosis-mediated antigen transfer to NK cells is NK-mediated killing of other NK cells, a process known as fratricide. In non-genetically modified NK cells trogocytosis of MHC class I-related chain A (MICA) in humans or retinoic acid early-inducible protein 1 (Rae-1) in mice has been shown to lead to fratricidal NK-mediated killing of NK cells (153, 154). This is also a common problem in CAR therapy, when the CAR target antigen is transferred from the tumor to the CAR-T or CAR-NK cell, leading to fratricide of CAR cells (145, 146). In the context of endogenous T or NK antigens like CD7 or CD38 respectively, knockout of the target antigen in the effector cell can overcome fratricidal killing (155, 156), but such an approach would not work for antigens which are transferred to effector cells *via* trogocytosis. As described above, design of the CAR to limit trogocytosis in CAR-T cells could not only improve antigen loss, but it can also prevent fratricide (147, 149, 152). In the case of NK-CARs, a recent elegant study used an inhibitory receptor targeting an NK-restricted antigen along with the tumour-targeted CAR in order to prevent trogocytosis and improve therapeutic activity (145). Future studies are needed to assess whether specific changes in the design of the various domains of the CAR construct can reduce trogocytosis mediated antigen loss and/or fratricide, and thereby improve the long-term efficacy of both CAR-T and CAR-NK therapies.

## Conclusion

While not yet as well understood as CAR-T, CAR-NK have shown promise in early studies treating both hematologic malignancies and solid tumors. However, despite the clear conceptual advantages to NK cell-based CAR therapies, and a robust clinical safety record for NK therapies in general, CAR-NK treatments have not had the strong efficacy and/or long-term persistence that are needed. The multiple reasons underlying the struggles for CAR-NK therapy have yet to be fully elucidated but certainly include many of the same challenges which limit CAR-T therapies. Hopefully by applying lessons learned over many years of experience in the CAR-T field, those working with CAR-NK cells will be able to capitalize on the unique assets offered by NK cell biology and create the next generation of revolutionary accessible and affordable cellular therapies.

## Author contributions

MK, DB, SL, MA, SM, and AV wrote, edited, and conceptualized the manuscript. All authors contributed to the article and approved the submitted version.
